# Why age categories in youth sport should be eliminated: Insights from performance development of youth female long jumpers

**DOI:** 10.3389/fphys.2023.1051208

**Published:** 2023-01-25

**Authors:** Eva Rüeger, Marie Javet, Dennis-Peter Born, Louis Heyer, Michael Romann

**Affiliations:** ^1^ Swiss Federal Institute of Sport Magglingen SFISM, Magglingen, Switzerland; ^2^ Swiss Swimming Federation, Ittigen, Switzerland; ^3^ Swiss Athletics Federation, Ittigen, Switzerland

**Keywords:** talent development, relative age effect, track and field, youth competition, longitudinal performance evaluation

## Abstract

Long-term sports participation and performance development are major issues in popular sports and talent development programs. This study aimed to provide longitudinal trends in youth female long jump performance development, participation, and relative age effects (RAEs), as longitudinal data for female athletes are missing. 51′894 season’s best results of female long jump athletes (*n* = 16′189) were acquired from the Swiss Athletics online database and analyzed within a range of 6–22 years of age. To examine longitudinal performance development and RAEs, data from athletes who participated in at least three seasons were selected (*n* = 41′253) and analyzed. Performance development was analyzed using age groups (AGs) and exact chronological age (CA) at competition. Differences between performances of birth quarters were analyzed using 83% confidence intervals (CIs) and smallest worthwhile change. Odds ratios (ORs) with 95% CI were used to quantify RAEs. With the traditional classification into age groups (AG), performances of athletes born between January and March (Q1) were significantly better than those born between October and December (Q4) from U8 to U17. Using exact CA resulted in similar performances in Q1 and Q4 until the U20 age category. The peak of participation was reached in the U12 category, and then decreased until the U23 category with a substantial drop at U17. Significant RAEs were observed from U8 to U19 and at U22. RAEs continuously decreased from U8 (large effect) to U14 (small effect). The present results show that differences in performance arise from the comparison of athletes in AGs. Thus, going beyond AGs and using exact CA, Q4 athletes could benefit from a realistic performance comparison, which promotes fair performance evaluation, un-biased talent development, realistic feedback, and long-term participation.

## Introduction

Understanding the pathway to athletic excellence remains a highly debated topic for stakeholders in sport (Côté et al., 2007; Gulbin et al., 2013). Performance development, along with participation and personal development, are the most important criteria for sustainable long-term talent development (Côté et al., 2007). However, performance potential cannot be predicted based on a single performance test, as long-term development is not linear and is influenced by the relative age effect (RAE) (Smith et al., 2018). To better evaluate performance potential, we should instead continuously assess athletes’ performances and create individualized performance curves that can be compared to the ideal trajectory of a specific development phase, while also taking the RAE into account.

The (RAE) describes the influence of the month of an athlete’s birth on their presence in the sport system and has been studied for almost 40 years (Barnsley et al., 1985; Smith et al., 2018). In predominantly physical sports, athletes born between January and March (first quartile, Q1) are over-represented, while those born between October and December (fourth quartile, Q4) are under-represented (Cobley et al., 2009; Smith et al., 2018). This can be explained by an age difference of up to 12 or 24 months between athletes in the same yearly or 2-year category, respectively (Helsen et al., 2005). Greater height and lean body mass are predictive of better physical capacities such as muscular strength, and speed, so in turn these characteristics provide physical performance advantages in most sport tasks (Viru et al., 1999; Malina et al., 2004). The relative age difference is greatest at birth and decreases exponentially during growth and is negligible in adulthood (Cobley et al., 2009). In team and individual sports, RAE affects both female and male athletes from the age of 4 years until adulthood (Cobley et al., 2009; Smith et al., 2018; Romann et al., 2018) and, can result in two major misjudgments in talent development: falsely supporting the more mature athletes born in Q1 who temporarily outperform their younger counterparts, but may have less performance potential; and the less advanced maturity of athletes born in Q4 may lead to deselection. As such, considering RAEs in talent identification and development systems could improve assessment of performance potential, support athletes’ long-term development, and improve equal participation opportunities.

Future performance is difficult to predict until the end of puberty due to growth, maturation and relative age, which are not reflected by a single measurement timepoint. Longitudinal analyses better represent the influence of these factors on performance development (Kraemer, 2000), and are needed to understand the long-term effects of RAE on athletes’ performance evolution and to identify individual and group development patterns. (Tan et al., 2012). Longitudinal approaches can also be used to investigate certain phenomena, such as the “underdog hypothesis”. This phenomenon suggests that later born athletes benefit from greater challenge during development because they are training and competing with relatively older peers. This leads to a relatively more frequent transition of later born athletes from junior into senior sport. (Gibbs et al., 2012; Kelly et al., 2020). However, the effect has so far only been shown in male athletes.

The prominent lack of female-specific research is well established. Although scientists are trying to rectify this, data on performance development in female athletes is sparce, particularly in track and field (Curran et al., 2019), and RAEs have mostly been investigated in cross-sectional studies (Cobley et al., 2009; Musch & Grondin, 2001; Smith et al., 2018). The limited RAE data in track and field show that RAE has an influence in most disciplines and in long jump the effect has been quantified as medium to large (Hollings et al., 2014; Romann & Cobley, 2015; Brazo-Sayavera et al., 2017; Boccia et al., 2021; Brustio et al., 2022). A recent study by Brustio et al. (2022) which investigated RAEs in 6827 female long jumpers showed medium effects in the 12–14-year age category and small effects in the 15–17-year age category. Similarly, previous analyses by Brustio et al. (2019) revealed medium and small RAEs in the top 100 U-18 and U-20 female long jump athletes, respectively. Thus, for the best possible interpretation of performance and results, the influence of the RAE on athletes’ pathways must be considered. However, information on the realistic developmental potential of female long jumpers—from the beginning to the end of puberty—is currently not available and can only be obtained using longitudinal data (Boccia et al., 2017). Given the need to optimize talent detection and development systems, in particular for female athletes, the aims of this study were 1) to statistically analyze the different performance curves as a function of birth quarter, age groups (AG) and exact chronological age (CA) in days, in order to shed light on the impact of RAEs on performance trajectories, 2) to analyze the RAE among female athletes participating in long jump during at least three seasons of competition.

## Methods

### Subjects

The Swiss system of talent identification, selection, and development is based on three levels of performance: a nationwide extracurricular program called Jugend und Sport (J + S), which is offered to all children; and regional and national Swiss talent development programs (Romann et al., 2018). Long jump is one of 77 sports available through the J&S program with 9584 (5646 female and 3938 male) licensed athletes in the U10 to U23 age categories (data from 2022). This includes only club and federation based practice. Selections are performed by the federation beginning at the age of 10 years (see Romann et al., 2018 for a more detailed description).

Data of all officially competitions licensed by the International Association of Athletics Federations were extracted from the Swiss Athletics online database for the years 2000–2019. Female athletes aged 6–22 years (*n* = 16′189) were selected so as to cover the full pathway of talent development. The athletes’ birth quarters were obtained from the dates of birth (Q1 = January to March, Q4 = October to December) and their exact chronological age (CA) at competition was calculated. As the current age categories cover a 2-years period, a fictitious annual grouping (AG) was made according to the age during the competition year to calculate the RAE on an annual basis.

### Procedure and data analysis

All results were filtered by AG and outliers were defined as results lower than −3 SD of the mean of each AG. The athletes’ best results within each AG they competed in were labelled as their seasons’ best (SB, *n* = 51′894). Athletes who participated in at least three seasons (*n* = 8′583) were included in the longitudinal performance analysis, resulting in 41′253 season’s best results. The number of athletes who did not participate for two consecutive seasons or more and who did not return to competition for more than 1 year were defined as dropout. For the calculation of the longitudinal performance development curves the data were organized as follows. In the data matrix, each row represented an athlete’s performance, as repeated measurements were recorded horizontally. A multivariate, longitudinal analysis was conducted to assess the development of long jump performance over time. To calculate the change in long jump performance over time, the exact age at which athletes competed was calculated based on the athletes’ date of birth. To determine the relationship between age and performance, the CA (i.e., age in years and days as independent variable) and the furthest jump performance (performance in m as dependent variable) were examined using a mixed model regression analysis. This model takes the correlation of intra-individual datapoints into account—as repeated measures of the same person are correlated (Tantular and Jaya, 2018) and was recently used in similar studies (Abbott et al., 2021; Brustio et al., 2022). Then the trend line of performance development was calculated using the model. CA was entered as a fixed factor, while participants were entered as a random factor. To present one fitted graph for the whole population, the “population level prediction” random effects (here only participants ID) were set to zero. Using this approach multiple R-squared was 0.68 for both the Q1 and the Q4 within the age categories model and 0.69 within the exact age model. The second degree polynomial function was chosen, as the second and multiple degree polynomial models did not differ significantly. (Abbott et al., 2021).

The longitudinal Q1 and Q4 performance development curves were plotted against AG and CA. The differences in performance development between curves were statistically analyzed within a 83% CI, which indicated if the Q1 and Q4 curves significantly differed (Austin & Hux, 2002). The smallest worthwhile change in performance differences between Q1 and Q4 were used to detect relevant effects (Hopkins et al., 1999). These estimates of smallest worthwhile changes in performance are useful thresholds for interpreting the magnitude of performance changes in athletes (Haugen & Buchheit, 2016). In this context, smallest worthwhile changes can be described as a small Cohen effect size. This effect size is calculated as 0.2 times the between-subject standard deviation within a specific population (Hopkins, 2000).

To quantify the RAE, odds ratios (OR) between Q1 and Q4, with a 95% confidence interval (95% CI), were calculated relative to the birthdate distribution of registered births among the Swiss population from 2010 to 2020 (Federal Statistical Office). OR were interpreted as effect sizes as follows: the RAE was significant if the CI did not include 1 and 1.00 ≤ OR < 1.22, 1.22 ≤ OR < 1.86, 1.86 ≤ OR < 3.00, and OR ≥ 3.00, were interpreted as negligible, small, medium and large, respectively (Olivier & Bell, 2013). If the OR was <1 and the CI did not include 1, the finding was interpreted as a significant inverse RAE. Inverse ORs <0.33 (1/3), 0.33 ≤ OR < 0.53 (1/1.86), 0.53 ≤ OR < 0.81, 0.81 ≤ OR < 1.0 were interpreted as large, medium, small, and negligible, respectively. All statistical analyses were performed in RStudio.

## Results

The three seasons’ of participation approach reduced the total number athletes included and seasons’ best results from 16′189 and 51,894 to 8′694 and 41,253, respectively: a loss of 20.5% of available results and 46.3% of athletes. The peak in participation was observed in the U12 category (*n* = 5,866) and decreased constantly thereafter. The maximum number of drop-outs was observed in the U17 category (*n* = 1,167, Table 1).

**TABLE 1:** RAEs of female long jumpers. Q1, Q4 = yearly quarters 1 and 4; OR = Odds ratio; CI = Confidence Interval. The number of athletes who did not participate for two consecutive seasons or more and who did not return to competition for more than 1 year were defined as dropout. H1 = first half year (Q1 + Q2).

Under U)	*n*	n drop-out	Q1 (%)	Q2 (%)	Q3 (%)	Q4 (%)	Or H1/H2	Or Q1/Q4	95% CI
U8	1654	-	40.8	30.4	18.0	10.9	2.5	3.8	(3.23, 4.48)
U9	2676	3	34.0	27.1	21.9	17.0	1.6	2.0	(1.81, 2.27)
U10	3936	14	32.4	26.7	23.1	17.8	1.4	1.9	(1.68, 2.03)
U11	5118	56	30.7	26.2	24.4	18.7	1.3	1.7	(1.54, 1.8)
U12	5866	143	29.9	26.2	24.4	19.5	1.3	1.6	(1.45, 1.68)
U13	5750	295	29.3	25.5	24.9	20.3	1.2	1.5	(1.36, 1.58)
U14	5328	424	28.2	25.6	25.3	20.9	1.2	1.4	(1.27, 1.48)
U15	4206	702	27.9	26.5	25.1	20.5	1.2	1.4	(1.27, 1.51)
U16	3166	670	26.9	26.7	25.4	21.0	1.2	1.3	(1.17, 1.44)
U17	1354	1167	26.3	27.3	24.9	21.5	1.2	1.2	(1.06, 1.45)
U18	861	370	28.6	27.3	24.3	19.9	1.3	1.5	(1.2, 1.78)
U19	529	244	27.6	27.2	25.7	19.5	1.2	1.4	(1.12, 1.85)
U20	329	167	25.8	28.9	23.7	21.6	1.2	1.2	(0.89, 1.67)
U21	219	77	31.5	23.7	24.2	20.6	1.2	1.6	(1.07, 2.27)
U22	156	101	27.6	25.6	25.0	21.8	1.1	1.3	(0.82, 2.01)
U23	105	-	22.9	23.8	30.5	22.9	0.9	1.0	(0.58, 1.79)
Total	8694	4433	30.0	26.4	24.2	19.4	1.3	1.6	(1.53, 1.62)

### Performance development

The performance development of athletes who participated in at least three seasons increased and reached a plateau in the U22 age category for Q1 athletes. However, the performance of Q4 athletes was increasing from U8 to U23.

**FIGURE 1:**
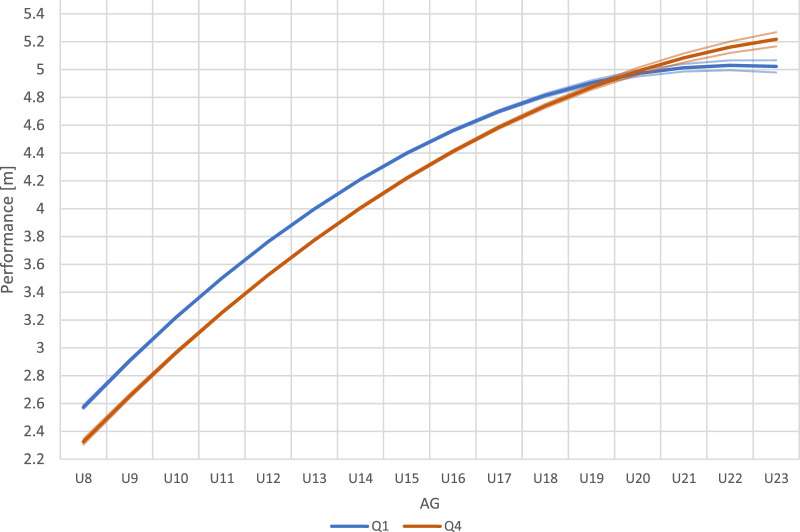
Long jump performance of Q1 and Q4 athletes with corresponding 83% confidence intervals in annual age groups (AG).

Smallest worthwhile change increased from 0.07 m in the U8 category to 0.09 m in the U23 category. The analysis showed that the differences in performance between Q1 and Q4 athletes, when compared by AG, were significant and relevant for the U8 to the U18 categories (Figure 1). Between U19 and U21 no relevant differences occurred. However, in U22 and U23 athletes of Q4 significantly outperformed Q1 athletes.

**FIGURE 2:**
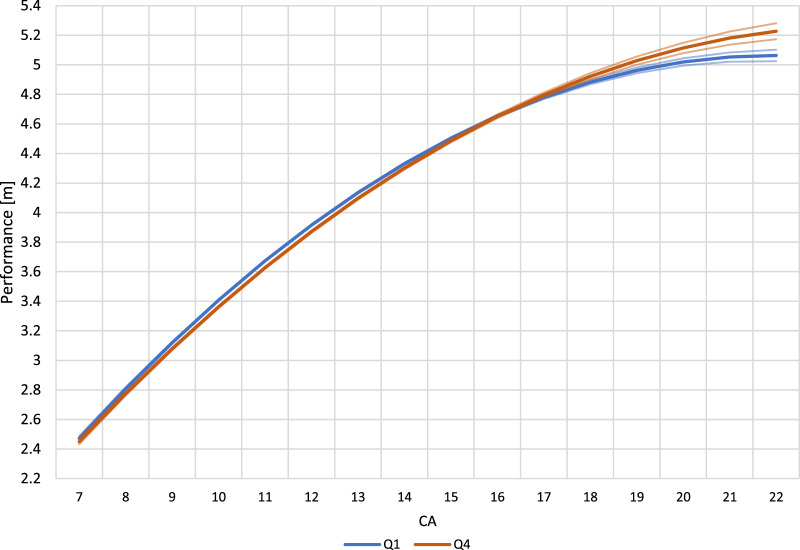
Long jump performance of Q1 and Q4 athletes with corresponding 83% confidence intervals using exact chronological age at competition (CA).

When the performance of Q1 and Q4 athletes were compared by CA, the difference in performance were statistically similar between U8 to U20 (Figure 2). Differences between curves were 0.01 m–0.02 m compared to the smallest worthwhile change of 0.07 m–0.09 m, which indicated no relevant difference between Q1 and Q4. However, Q4 athletes demonstrated significantly better performances in the U21 to the U23 categories.

### Relative age effect

An overall RAE with a small effect was observed [OR 1.57 (95% CI 1.53, 1.62)] among female athletes participating in long jump competitions over at least three seasons. The RAE was significant from U8 to U19 and in U21. It decreased continuously from U8, where a large effect [OR 3.80 (95% CI 3.23, 4.48)] was observed, to U14, which presented a small effect [OR 1.37 (CI 1.27, 1.48)]. The effect remained small until U19 and ranged from small to insignificant from U20 to U23 (Table 1).

## Discussion

The goal of this study was to analyze RAEs and their impact on performance trajectories in female long jumpers who competed for at least three consecutive years. We managed to illustrate the evolution of performance with age in female long jumpers and to highlight the significant effects of RAEs in this discipline. The main results of this study are firstly that the creation of age categories causes a performance gap between athletes born in Q1 and those born in Q4, which disappears when the performances are compared using exact CA. From the age of 21 (comparison by CA) or from the U22 category (comparison by AGs) onwards, Q4 athletes outperformed Q1 athletes.

### Time course of RAE

In youth sport, the creation of age categories is necessary to organize competitions that minimize age differences and make performance comparisons possible. However, as soon as categories are created, the RAE inevitably appears. Its effects have been studied for several years and are gradually being understood in terms of their influence on athlete participation and talent identification and development. One aspect that is not yet well researched is the difference of the RAE in male and female athletes and its longitudinal development. Votteler and Höner (2017) showed that RAE was driven by the entry of new soccer players into the systems, rather than by the selection of already present soccer players to higher levels. They also showed a trend of increasing RAE up to the U15 category, which decreased in older age categories. In the results of the present study, which is based on an individual discipline and not a team sport, prolonged participation (3 or more seasons) showed a decrease in RAE starting at the age of U9 (OR 3.8 in U8 vs. OR 2.0 in U9). More specifically, the data from this study showed that RAE peaked at U8 (strong effect) and then decreased steadily until U17 (small effect). Thus, differentiation between sports seems to be necessary given the differences in RAE development (Baker et al., 2009; Romann et al., 2018). As athletics is primarily based on purely physical skills such as strength, speed and/or endurance (Kearney et al., 2018), the RAE profile in long jump can be explained by the physical superiority of Q1 athletes until the end of puberty. However, research on AG younger than U15 is sparse, and more studies are needed to elucidate the origin of RAE (Romann et al., 2020). Furthermore, from the onset of puberty, growth and maturation should also be considered as additional factors in the performance of young athletes (Duarte et al., 2019; Radnor et al., 2021).

Girls reach peak growth aged 12.0 years, which is approximately 2 years earlier than boys (Syrjälä et al., 2021). Therefore, and due to the lack of data on female athletes, female RAE development should be studied separately.

### Longitudinal performance and implication in talent identification and development

Previous studies have linked RAEs to dropouts (Delorme et al., 2011; Lemez et al., 2014) and tried to identify their origins (Romann et al., 2020): By being relatively older, athletes may receive more support and interest from coaches, thus, they improve and are selected, while relatively younger athletes are often more neglected during training and competition and receive less positive feedback, which in turn can create a vicious cycle leading to dropout. However in line with the underdog hypothesis, Q4 athletes who make it through the selection processes have been reported to outperform their counterparts at adult age (Gibbs et al., 2012; McCarthy et al., 2016; Kelly et al., 2020). The more difficult conditions experienced during their training and competition may allow them to develop greater technical and psychological skills, which may explain their better performance once the physical differences to Q1 athletes are overcome (Carling et al., 2009). The results of this study support this hypothesis. At the age of 17 a maximum dropout is reached and Q4 athletes start outperforming Q1 athletes. The RAE reduces and even becomes negligible from this age on; and Q1 athletes that are caught up by Q4 seem to stop participating in competitions. However, drop out is a complex and important topic and further research is needed.

Avoiding RAEs throughout athletes’ career paths can make talent identification and development systems more efficient and place resources in the right place and moment. In the current system, many athletes are promoted only because of their age (and/or developmental) advantage. This means that resources are used inefficiently and there is no equality of opportunity in talent development. Specifically longitudinal tracking of RAEs could help adjust the timing of selections and make talent support structures more effective. According to Hollings et al. (2014); Boccia et al. (2021), some relatively younger athletes dropout of their sport before reaching their full potential, while relatively older athletes reach a performance plateau at an early age. This could lead to a decline in overall performance within the talent pool. Additionally recent research has shown that RAEs have a consistent effect on participation in children’s football at the grassroots level. To protect young athletes from discrimination, RAE biases should be analyzed and eliminated at all stages of sport participation and selection. Modifications to the organizational structure of sport participation, athlete development systems and coach education are recommended to prevent RAE-related inequalities (Romann et al., 2020).

According to AGs and CA, the difference in performance curves between Q1 and Q4 show that in the current long jump system in Switzerland, the comparison of Q1 and Q4 athletes’ performances is biased. Moreover, the categories are generally organized over 2 years or more, which further accentuates the differences in relative age. Thus, the shift in performance curves when chronological age is considered provides important information on the performance of Q4 athletes. These findings would also support the introduction of corrective adjustment procedures (CAPs) based on age categories (Romann & Cobley, 2015; Brustio et al., 2022). When using CAPs, the mean expected performance curve is calculated. In a second step, one can calculate an expected value for each athlete and compare it with the current performance. This allows a performance comparison considering the exact chronological age of the athlete (Romann & Cobley, 2015).

### Limitations

All analyses are based on Swiss long jump athletes. Applying the results to other sports should be done with caution. Hence, more studies evaluating longitudinal RAE development on talent selection and development in other sports are desirable. Furthermore, selection levels and age groups should be taken into account when analyzing talent development, as they influence RAE. It is important to note that maturation has a significant influence on performance at an intra- and inter-individual level (Malina et al., 2004; Radnor et al., 2021). This aspect could not be included in this study and, therefore, should be subject of future studies. Nevertheless, this is the first study to analyze a nationwide database of female athletes longitudinally across all age categories and selection levels in youth long jump. Therefore, this study highlights the evolution of RAEs over age categories, and how selections may influence participation. Future research should include growth and maturation in the evaluation of longitudinal performance and dropout analysis.

### Practical implications

Practitioners, such as coaches and staff, should consider and be aware that a) RAEs exist in female long jump; b) performances in female long jump are biased until the U18 age category if a “classic” age category approach is applied; c) RAEs can be removed up until the U20 age category if an exact age approach is used. For example, the data from this study shows that in the current system, female athletes born late in the year (Q4) of an age category are systematically disadvantaged. By comparing performances by exact age on the day of competition, relative age differences could be eliminated. The same approach has already been successfully tested and applied in athletics sprint and swimming (Romann & Cobley, 2015; Abbott et al., 2021; Brustio et al., 2022). This improvement in equality of opportunity and the associated reduction in RAEs could result in fewer young athletes being wrongly de-selected. Bringing all aspects together, practitioners should apply an “exact age” approach using longitudinal data to evaluate the performance of youth female athletes. This could be implemented in the long jump competition, for example, by calculating the expected value for each athlete’s exact age. An age-adjusted ranking can then be created from the difference between the achieved performance and the expected value.

## Conclusion

Given the need to optimize sports participation, talent identification and development systems for female athletes, the present study—including 41′253 female long jump results—underlines the differences in performance that arise from a comparison of athletes in AGs and subsequently leads to RAEs. This means that in Swiss female long jump many athletes are promoted only because of their age (and/or developmental) advantage, that resources are used inefficiently and that there is no equality of opportunity in talent development.

When using an exact chronological age approach, relative age differences in performances can be eliminated up until the U20 AG. However, it should be noted that in Swiss female long jump the RAE leads to better performances in Q4 athletes as they approach adulthood, illustrating the underdog hypothesis. This new, data driven approach may improve performance evaluation and could lead to more effective talent identification and talent development in the sport system. Additionally, it may allow fair performance evaluation, realistic feedbacks, and long-term sport participation for young athletes.

## Data Availability

Publicly available datasets were analyzed in this study. This data can be found here: https://www.swiss-athletics.ch/de/bestenliste/.
